# Possible role of the gut microbiota–brain axis in the antidepressant effects of (*R*)-ketamine in a social defeat stress model

**DOI:** 10.1038/s41398-017-0031-4

**Published:** 2017-12-18

**Authors:** Chun Yang, Youge Qu, Yuko Fujita, Qian Ren, Min Ma, Chao Dong, Kenji Hashimoto

**Affiliations:** 1 Division of Clinical Neuroscience, Chiba University Center for Forensic Mental Health, Chiba, Japan; 2 Present Address: Department of Anesthesiology, Tongji Hospital, Tongji Medical College, Huazhong University of Science and Technology, Wuhan, 430030 China

## Abstract

Accumulating evidence suggests that the gut microbiota–brain axis plays a role in the pathogenesis of depression, thereby contributing to the antidepressant actions of certain compounds. (*R*)-ketamine has a greater potency and longer-lasting antidepressant effects than (*S*)-ketamine. Here, we investigated whether the gut microbiota plays a role in the antidepressant effects of these two ketamine enantiomers. The role of the gut microbiota in the antidepressant effects of ketamine enantiomers in a chronic social defeat stress (CSDS) model of depression was examined using 16S ribosomal RNA gene sequencing of fecal samples. At the phylum level, CSDS-susceptible mice showed alterations in the levels of *Tenericutes* and *Actinobacteria*; however, neither ketamine enantiomers influenced these alterations. At the class level, both ketamine enantiomers significantly attenuated the increase in the levels of *Deltaproteobacteria* in the susceptible mice after CSDS. Furthermore, (*R*)-ketamine, but not (*S*)-ketamine, significantly attenuated the reduction in the levels of *Mollicutes* in the susceptible mice. At the genus level, both ketamine enantiomers significantly attenuated the decrease in the levels of *Butyricimonas* in the susceptible mice. Notably, (*R*)-ketamine was more potent than (*S*)-ketamine at reducing the levels of *Butyricimonas* in the susceptible mice. In conclusion, this study suggests that the antidepressant effects of two enantiomers of ketamine in CSDS model may be partly mediated by the restoration of the gut microbiota. Furthermore, the specific effect of (*R*)-ketamine on the levels of *Mollicutes* and *Butyricimonas* may explain its robust antidepressant action.

## Introduction

In 2000, Berman et al.^1^ reported that a subanesthetic dose of ketamine, an *N*-methyl-D-aspartate receptor (NMDAR) antagonist, elicits rapid and sustained antidepressant effects in depressed patients. Subsequent clinical studies replicated ketamine’s antidepressant effects in treatment-resistant major depression and treatment-resistant bipolar depression^2–5^. Furthermore, recent meta-analyses confirmed that ketamine exhibits rapid and sustained antidepressant effects in treatment-resistant depressed patients^6,7^. Interestingly, ketamine demonstrated a rapid reduction of suicidal ideation in treatment-resistant depressed patients^8–10^. However, it is well recognized that ketamine produces acute psychotomimetic side effects after single or repeated infusions^1,2,4,5,11,12^. Therefore, while ketamine is the most prominent antidepressant for treatment-resistant depression^13–18^, its psychotomimetic side effects and potential for abuse should not be ignored^19–23^.

Ketamine is a racemic mixture comprising equal parts of (*R*)-ketamine and (*S*)-ketamine. (*S*)-ketamine exhibits an approximately threefold to fourfold greater binding affinity for NMDARs than (*R*)-ketamine, which pharmacologically explains why (*S*)-ketamine has an approximately fourfold greater anesthetic potency and greater undesirable psychotomimetic side effects than (*R*)-ketamine^16–18,24^. Several groups, including our laboratory, demonstrated that (*R*)-ketamine has more potent and longer-lasting antidepressant effects than (*S*)-ketamine in animal models of depression^25–30^. Unlike (*S*)-ketamine, (*R*)-ketamine appears to lack psychotomimetic side effects and potential for abuse^26,31,32^. However, the precise mechanisms underlying the antidepressant actions of ketamine enantiomers remain unclear.

The gut microbiota–brain axis is a complex multiorgan bidirectional signaling system between the gut microbiota and brain that plays a crucial role in host physiology, homeostasis, development, and metabolism^33–35^. Several studies suggest that the gut microbiota contributes to the pathogenesis of depression and the antidepressant actions of certain compounds^36–45^. Therefore, the present study examined whether the gut microbiota plays a role in the mechanisms underlying the antidepressant actions of (*R*)-ketamine and (*S*)-ketamine in a chronic social defeat stress (CSDS) model of depression.

## Materials and methods

### Animals

Male adult C57BL/6 mice, aged 8 weeks (body weight 20–25 g, Japan SLC Inc., Hamamatsu, Japan) and male adult CD1 (ICR) mice, aged 13–15 weeks (body weight >40 g, Japan SLC Inc.) were used. Animals were housed under controlled temperatures and 12 h light/dark cycles (lights on between 0700–1900 hours), with ad libitum food (CE-2; CLEA Japan Inc., Tokyo, Japan) and water. This study was carried out in strict accordance with the recommendations in the Guide for the Care and Use of Laboratory Animals of the National Institutes of Health, USA. The protocol was approved by the Chiba University Institutional Animal Care and Use Committee.

### Materials

(*R*)-ketamine hydrochloride and (*S*)-ketamine hydrochloride were prepared by recrystallization of (*R,S*)-ketamine (Ketalar^®^, (*R,S*)-ketamine hydrochloride, Daiichi Sankyo Pharmaceutical Ltd., Tokyo, Japan) and D-(-)-tartaric acid (or L- ( + )-tartaric acid), as reported previously^25^. The purity of two ketamine enantiomers was determined by a high-performance liquid chromatography (CHIRALPAK^®^ IA, Column size: 250 × 4.6 mm, Mobile phase: n-hexane/dichloromethane/diethylamine (75/25/0.1), Daicel Corporation, Tokyo, Japan). The contamination of another enantiomer was not detected for two ketamine enantiomers. The dose (10 mg/kg as ketamine hydrochloride) of (*R*)-ketamine and (*S*)-ketamine was used as previously reported^25–31^.

### CSDS model

The procedure of CSDS was performed as reported previously^26,29,30,46–50^. The C57BL/6 mice were exposed to a different CD1 aggressor mouse for 10 min/day, total for 10 days. When the social defeat session ended, the resident CD1 mouse and the intruder mouse were housed in one half of the cage separated by a perforated Plexiglas divider to allow visual, olfactory, and auditory contact for the remainder of the 24-h period. Subsequently, all mice were housed individually 24 h after the last session. On day 11, social interaction test (SIT) was performed to select subgroups of mice that were susceptible and unsusceptible to social defeat stress. This was accomplished by placing mice in an interaction test box (42 × 42 cm) with an empty wire-mesh cage (10 × 4.5 cm) located at one end. The movement of the mice was tracked for 2.5 min, followed by 2.5 min in the presence of an unfamiliar aggressor confined in the wire-mesh cage. The duration of the subject’s presence in the “interaction zone” (defined as the 8-cm-wide area surrounding the wire-mesh cage) was recorded by a stopwatch. The interaction ratio was calculated as time spent in an interaction zone with an aggressor/time spent in an interaction zone without an aggressor. An interaction ratio of 1 was set as the cutoff: mice with scores <1 were defined as “susceptible” to social defeat stress and those with scores ≥1 were defined as “unsusceptible”. In the experiments, ~70–80 % of mice were susceptible after CSDS. Susceptible mice were randomly divided into the subsequent experiments. Control mice without social defeat stress were housed in the same cage before the behavioral tests.

### Treatment and behavioral tests

**Fig. 1:**
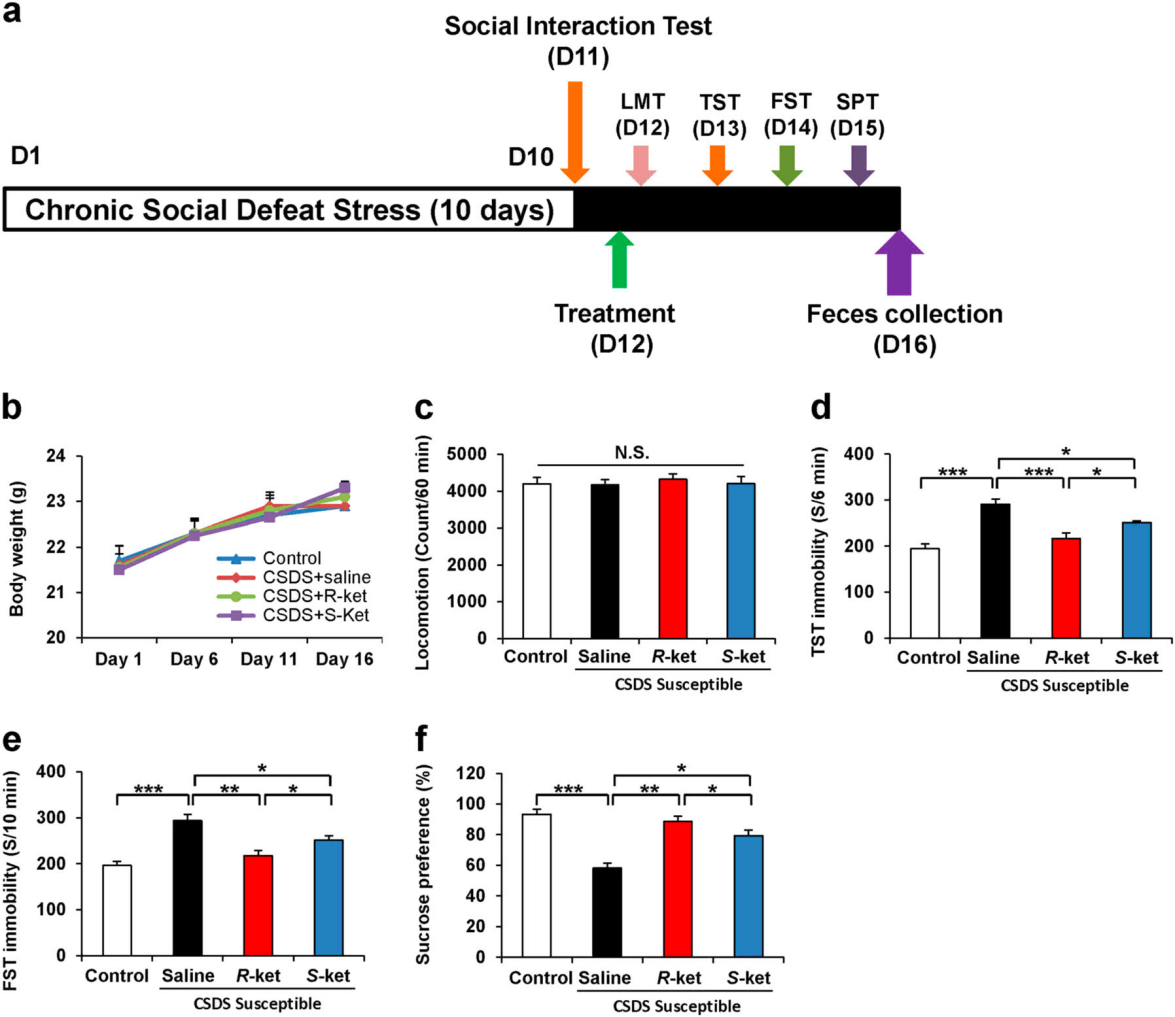
Antidepressant effects of ketamine enantiomers in susceptible mice after CSDS **a** The schedule of CSDS model, treatment, behavioral tests, and feces collection. CSDS was performed from day 1 to day 10, and social interaction test (SIT) was performed on day 11. Saline, (*R*)-ketamine, or (*S*)-ketamine were administered i.p. into CSDS-susceptible mice. Behavioral tests and SPT were performed from day 12 to day 15. On day 16, mouse feces was collected. **b** Body weight (time: F_3,15_ = 9.3, *P* < 0.001, treatment: F_3,15_ = 0.268, *P* = 0.848, interaction: F_9,15_ = 0.576, *P* = 0.813). **c**–**e** Behavioral tests including locomotion test (LMT; one-way ANOVA, F_3,20_ = 0 159, *P* = 0.923), TST (F_3,20_ = 18.362, *P* < 0.001) and FST (F_3,20_ = 15.107, *P* < 0.001) were performed after treatment. **f** SPT was performed 3 days after treatment (F_3,20_ = 20.287, *P* < 0.001). Data are shown as mean ± SEM (*n* = 6). *P < 0.05, **P < 0.01, ***P < 0.001. *NS* not significant

Saline (10 ml/kg), (*R*)-ketamine (10 mg/kg), or (*S*)-ketamine (10 mg/kg) was administered intraperitoneally (i.p.) into the susceptible mice after CSDS. Saline (10 ml/kg) was also administered i.p. into control mice (Fig. 1a). Behavioral tests, including locomotion test, tail suspension test (TST), forced swimming test (FST), and 1% sucrose preference test (SPT), were performed as reported previously^26,29,30,46–50^. Behavioral tests were also performed by two observers who were blinded to the group assignment of mice. Each treatment group was equally represented in each experimental cohort.

#### Locomotion

The locomotor activity of mice was measured by an animal movement analysis system SCANET MV-40 (MELQUEST Co., Ltd., Toyama, Japan). The mice were placed in experimental cages (length × width × height: 560 × 560 × 330 mm). The cumulative exercise was recorded for 60 min. Cages were cleaned between testing session.

#### TST

A small piece of adhesive tape was placed ~2 cm from the tip of the tail for mouse. A single hole was punched in the tape and mice were hung individually on a hook. The immobility time was recorded for 10 min. Mice were considered immobile only when they hung passively and completely motionless.

#### FST

The FST was tested by an automated forced-swim apparatus SCANET MV-40 (MELQUEST Co., Ltd., Toyama, Japan). The mice were placed individually in a cylinder (diameter: 23 cm; height: 31 cm) containing 15 cm of water, maintained at 23 ± 1℃. Immobility time from activity time as (total) − (active) time was calculated by the apparatus analysis software. The immobility time for mouse was recorded for 6 min.

#### SPT

Mice were exposed to water and 1% sucrose solution for 48 h, followed by 4 h of water and food deprivation and a 1 h exposure to two identical bottles, one is water, and another is 1% sucrose solution. The bottles containing water and sucrose were weighed before and at the end of this period. The sucrose preference was calculated as a percentage of sucrose solution consumption to the total liquid consumption.

### 16S rRNA analysis of fecal samples

The fecal samples were collected 4 days (day 16) after a single dose of saline (10 ml/kg), (*R*)-ketamine (10 mg/kg), or (*S*)-ketamine (10 mg/kg). They placed in 1.5 ml tubes, snap-frozen on dry ice, and stored at −80°C. The 16S rRNA analysis of fecal samples was performed at Takara Bio. Inc. (Shiga, Japan). The DNA extraction was performed using the MoBio Powerlyzer Powersoil DNA Isolation Kit (MoBio Laboratories, Carlsbad, CA, USA). The V4 hypervariable region of the bacterial 16S rRNA gene was amplified from the fecal DNA extracts using modified universal bacterial primer pairs 515 F (5′-TCGTCGGCAGCGTCAGATGTGTATAAGAGACAGGTGCCAGCMGCCGCGGTAA-3′) and 806 R (5′-GTCTCGTGGGCTCGGAGATGTGTATAAGAGACAGGGACTACHVGGGTWTCTAAT-3′) with Illumina adaptor overhang sequences. Amplicons were generated, cleaned, indexed, and sequenced according to the Illumina MiSeq 16S Metagenomic Sequencing Library Preparation protocol (http://support.illumina.com/ downloads/16s_metagenomic_sequencing_library_preparation.html) with slight modifications. Sequencing data were combined and sample identification assigned to multiplexed reads using the MOTHUR software environment^42,51^. The data were denoised; low-quality sequences, pyrosequencing errors, and chimeras were removed, and then sequences were clustered into operational taxonomic units (OTUs) at 97% identity using the CD-HITOTU pipeline (available from http://eeizhong-lab.ucsd.edu/cd-hit-otu)^42,52^. OTUs containing fewer than four reads per individual diet/animal combination were excluded due to the likelihood of there being a sequencing artifact. The samples were normalized by randomly resampling sequences used to the lowest number of sequences per sample (each diet/animal combination) using Daisychopper (http://www.festinalente.me/bioinf/). Taxonomic classification of OTUs was conducted using the Ribosomal Database Project Classifier^42,53^.

### Statistical analysis

The data show as the mean ± SEM. Analysis was performed using PASW Statistics 20 (formerly SPSS Statistics; SPSS, Tokyo, Japan). The data were analyzed using the one-way analysis of variance (ANOVA) or two-way ANOVA, followed by post hoc Tukey test. Furthermore, Principal Coordinate Analysis (PCoA) was performed to visualize similarities or dissimilarities of the data of four groups. The *P* values of less than 0.05 were considered statistically significant.

## Results

### (*R*)-ketamine showed more potent antidepressant effects than (*S*)-ketamine in the susceptible mice after CSDS

The antidepressant effects of (*R*)-ketamine and (*S*)-ketamine in the susceptible mice after CSDS were examined (Fig. 1a). There were no changes in the body weight among the four groups (Fig. 1b). There were no significant differences in the locomotion among the four groups (Fig. 1c). In the TST and FST, both (*R*)-ketamine and (*S*)-ketamine significantly decreased the increased immobility time in the susceptible mice after CSDS (Figs. 1d, 1e). Furthermore, (*R*)-ketamine showed greater antidepressant effects than (*S*)-ketamine. In the SPT, (*R*)-ketamine exerted more potent anti-anhedonia effects than (*S*)-ketamine (Fig. 1f). These data indicate that (*R*)-ketamine exerts more potent antidepressant and anti-anhedonia effects than (*S*)-ketamine, consistent with our previous reports^26,29,30^.

### The PCoA analysis of the gut bacterium data

**Fig. 2:**
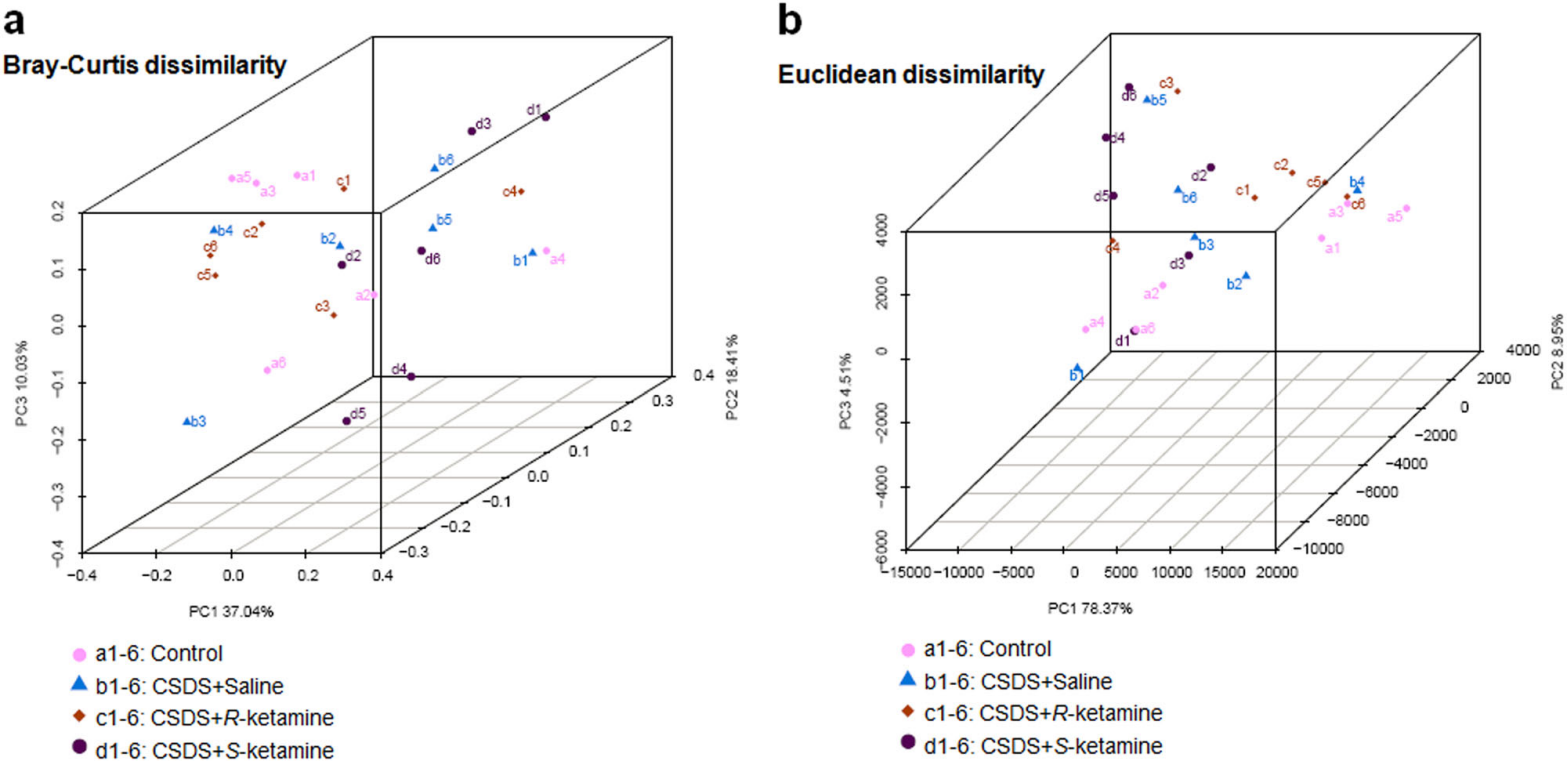
The PCoA analysis of the gut bacterium data **a** The PCoA analysis plots of Bray–Curtis dissimilarity among the four groups. **b** The PCoA analysis plots of Euclidean dissimilarity among the four groups

The PCoA analysis plots of Bray–Curtis dissimilarity among the four groups showed that the dots of control group (a1–a6) were close to the dots of (*R*)-ketamine-treated group (c1–c6) compared with (*S*)-ketamine-treated group (d1–d6; Fig. 2a). Furthermore, the PCoA analysis plots of Euclidean dissimilarity showed that the dots of control group (a1–a6) were close to the dots of (*R*)-ketamine-treated group (c1–c6) compared with (*S*)-ketamine-treated group (d1–d6; Fig. 2b). Thus, it is likely that (*R*)-ketamine has more potency to improve the altered composition of gut microbiota after CSDS than (*S*)-ketamine.

### Altered composition in the gut bacterium at the phylum level

**Fig. 3:**
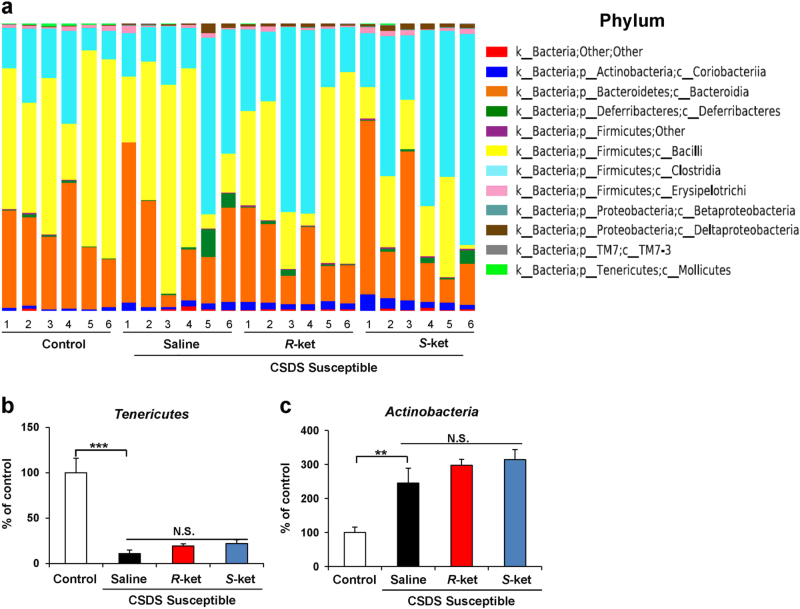
Altered composition in the gut bacterium at the phylum levels **a** The phylum levels of gut bacterium 4 days after a single dose of saline, (*R*)-ketamine, or (*S*)-ketamine. **b** The phylum levels of *Tenericutes* (one-way ANOVA: F_3,20_ = 23.58, *P* < 0.001) in the gut. **c** The phylum levels of *Actinobacteria* (one-way ANOVA: F_3,20_ = 11.39, *P* < 0.001) in the gut. Data are shown as mean ± SEM (*n* = 6). **P < 0.01, ***P < 0.001. *NS* not significant

The phylum levels of gut bacterium 4 days (day 16) after a single dose of saline, (*R*)-ketamine, or (*S*)-ketamine are shown (Fig. 3a). *Tenericutes* in the gut were significantly decreased in the susceptible mice after CSDS, although neither ketamine enantiomers affected the decreased levels of *Tenericutes* in the susceptible mice (Fig. 3b). Furthermore, the susceptible mice had the increased levels of *Actinobacteria*, although neither ketamine enantiomers affected the increased levels of *Actinobacteria* in the susceptible mice (Fig. 3c).

### Altered composition in the gut bacteria at the class level

**Fig. 4:**
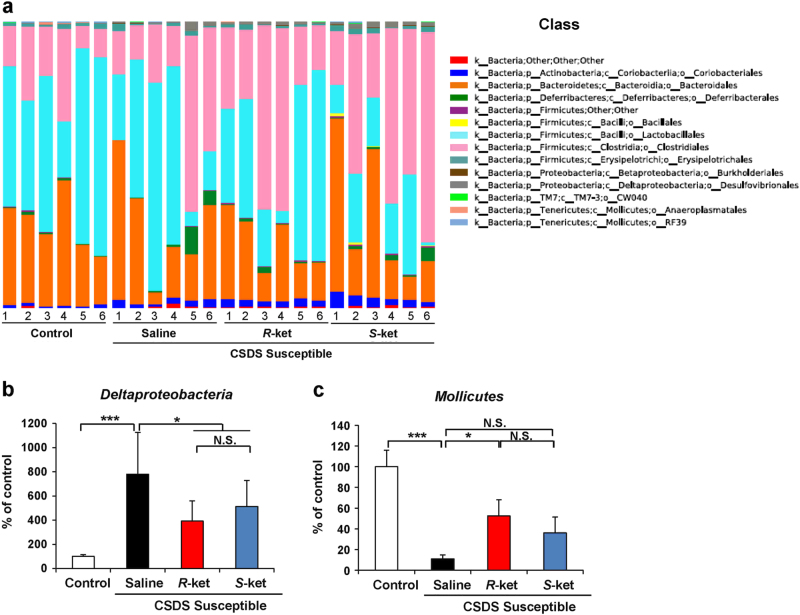
Altered composition in the gut bacteria at the class levels **a** The class levels of gut bacterium 4 days after a single dose of saline, (*R*)-ketamine, or (*S*)-ketamine. **b** The class levels of *Deltaproteobacteria* (one-way ANOVA: F_3,20_ = 9.692, *P* < 0.001) in the gut. **c** The class levels of *Mollicutes* (one-way ANOVA: F_3,20_ = 7.480, *P* = 0.002) in the gut. Data are shown as mean ± SEM (*n* = 6). *P < 0.05, ***P < 0.001. *NS* not significant

The class levels of gut bacterium 4 days after a single dose of saline, (*R*)-ketamine, or (*S*)-ketamine are shown (Fig. 4a). *Deltaproteobacteria* were significantly increased in the susceptible mice after CSDS. Both (*R*)-ketamine and (*S*)-ketamine significantly decreased the reduced levels of *Deltaproteobacteria* in the susceptible mice (Fig. 4b). Furthermore, *Mollicutes* were significantly decreased in the susceptible mice after CSDS. Interestingly, (*R*)-ketamine, but not (*S*)-ketamine, significantly attenuated the reduced levels of *Mollicutes* in the susceptible mice (Fig. 4c).

### Altered composition in the gut bacteria at the family level

**Fig. 5:**
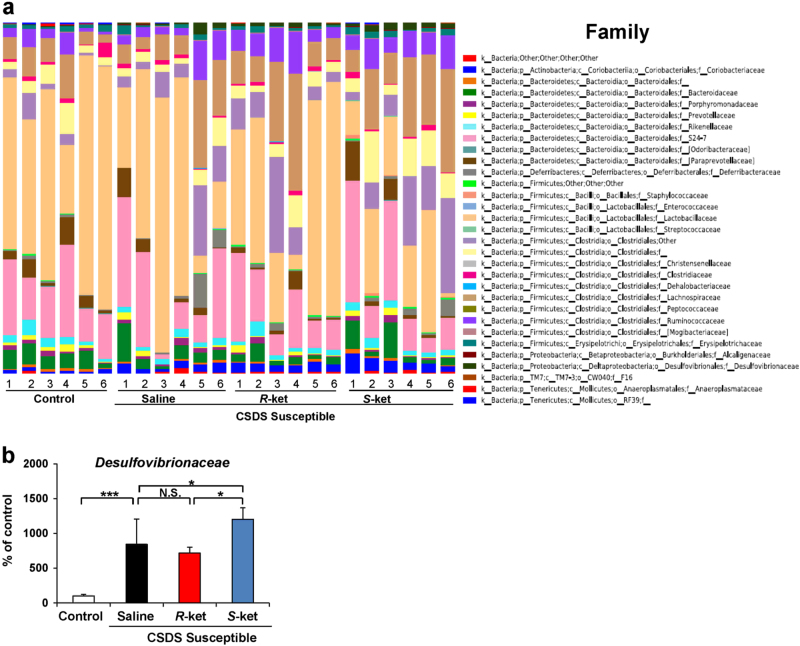
Altered composition in the gut bacteria at the family levels **a** The family levels of gut bacterium 4 days after a single dose of saline, (*R*)-ketamine, or (*S*)-ketamine. **b** The family levels of *Desulfovibrionaceae* (one-way ANOVA: F_3,20_ = 5.103, *P* = 0.009) in the gut. Data are shown as mean ± SEM (*n* = 6). *P < 0.05, ***P < 0.001. *NS* not significant

The family levels of gut bacterium 4 days after a single dose of saline, (*R*)-ketamine, or (*S*)-ketamine are shown (Fig. 5a). *Desulfovibrionaceae* were significantly increased in the susceptible mice after CSDS. Furthermore, (*S*)-ketamine significantly enhanced the increased levels of *Desulfovibrionaceae* in the susceptible mice, although (*R*)-ketamine did not alter the increased levels of *Desulfovibrionaceae* (Fig. 5b).

### Altered composition in the gut bacteria at the genus level

**Fig. 6:**
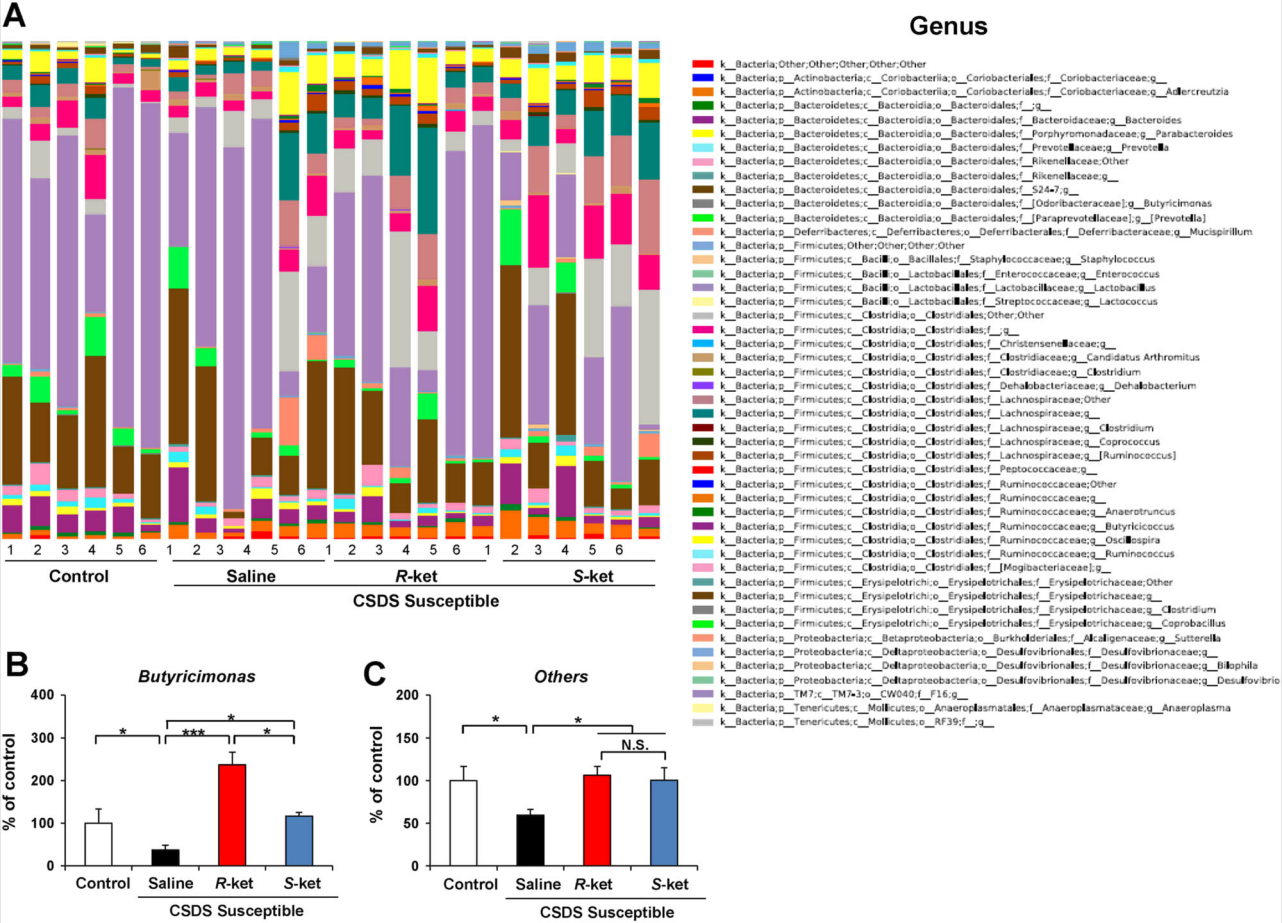
Altered composition in the gut bacteria at the genus levels **a**: The genus level of gut bacterium 4 days after a single dose of saline, (*R*)-ketamine or (*S*)-ketamine. **b**: The genus levels of *Butyricimonas* (one-way ANOVA: F_3,20_ = 3.177, *P* = 0.046) in the gut. **c**: The genus levels of other bacterium (one-way ANOVA: F_3,20_ = 3.54, *P* = 0.033) in the gut. Data are shown as mean ± SEM (*n* = 6). *P < 0.05, ***P < 0.001. *NS* not significant

The genus levels of gut bacterium 4 days after a single dose of saline, (*R*)-ketamine, or (*S*)-ketamine are shown (Fig. 6a). *Butyricimonas* were significantly decreased in the susceptible mice after CSDS. Furthermore, both (*R*)-ketamine and (*S*)-ketamine significantly increased the reduced levels of *Butyricimonas* in the susceptible mice. In addition, (*R*)-ketamine was more potent than (*S*)-ketamine (Fig. 6b). Moreover, both (*R*)-ketamine and (*S*)-ketamine significantly increased the reduced levels of others in the susceptible mice (Fig. 6c).

## Discussion

The levels of *Tenericutes*, a phylum of bacteria, were significantly decreased and the levels of *Actinobacteria*, a phylum of Gram-positive bacteria, were significantly increased in the susceptible mice after CSDS. Neither of the ketamine enantiomers had an effect on these alterations. In contrast, both ketamine enantiomers significantly attenuated the increased levels of *Deltaproteobacteria*, a class of *Proteobacteria*, in the susceptible mice. Interestingly, (*R*)-ketamine, but not (*S*)-ketamine, significantly increased the reduced levels of *Mollicutes* in the susceptible mice. We also observed that (*S*)-ketamine, but not (*R*)-ketamine, significantly enhanced the increased levels of *Desulfovibrionaceae*, a family of *Proteobacteria*, in the susceptible mice. Lastly, while both ketamine enantiomers significantly attenuated the reduced levels of the genus *Butyricimonas* in the susceptible mice, (*R*)-ketamine was more potent than (*S*)-ketamine. These findings suggest that an altered gut microbiota composition plays a role in the depression-like phenotype of CSDS-susceptible mice and that the restoration of the gut microbiota induced by (*R*)-ketamine partly explains its robust antidepressant effects.

At the phylum level, levels of *Actinobacteria* were significantly higher in active major depressive disorder (MDD) patients than in healthy control subjects^36,38^. Consistent with these results, we found increased levels of *Actinobacteria* in CSDS-susceptible mice. Although the exact physiological implications of *Actinobacteria* in depression are not fully understood, it is likely that increased levels of *Actinobacteria* in the gut contribute to the pathogenesis of depression. However, neither of the ketamine enantiomers reduced the levels of *Actinobacteria* in CSDS-susceptible mice.

At the class level, the levels of *Deltaproteobacteria* and *Mollicutes* were altered in CSDS-susceptible mice. There are no available reports on altered levels of *Deltaproteobacteria* and *Mollicutes* in MDD patients. However, increased levels of sulfite-reducing *Deltaproteobacteria* were found in ulcerative colitis (UC) patients with higher levels of inflammation^54^. Given the role of inflammation in CSDS-susceptible mice^42^, it is possible that increased levels of *Deltaproteobacteria* contribute to the depression-like phenotype of these mice through inflammatory responses. In addition, the reduction of levels of *Deltaproteobacteria* in CSDS-susceptible mice treated with either of the ketamine enantiomers may partly explain their antidepressant actions. Interestingly, (*R*)-ketamine, but not (*S*)-ketamine, significantly attenuated the reduced levels of *Mollicutes* in CSDS-susceptible mice. Although the precise physiological implications of *Deltaproteobacteria* and *Mollicutes* in depression are unknown, it is likely that changes in the level of these bacteria induced by (*R*)-ketamine partially mediate its antidepressant action. Nonetheless, further studies on the relationship between (*R*)-ketamine’s antidepressant effects and *Deltaproteobacteria* and *Mollicutes* are needed.

At the family level, we observed an increase in the levels of *Desulfovibrionaceae* in CSDS-susceptible mice. *Desulfovibrionaceae* are sulfate-reducing, nonfermenting, anaerobic, Gram-negative bacteria characterized by the presence of desulfoviridine^55^. *Desulfovibrionaceae* in the gut are the main biological source of hydrogen sulfate (H_2_S), which regulates a wide range of physiological functions, including the cardiovascular, neuronal, immune, respiratory, gastrointestinal, liver, and endocrine systems, by influencing cellular signaling pathways and sulfhydration of target proteins^56,57^. Furthermore, the levels of *Desulfovibrionaceae* were higher in animal models of metabolic syndrome^58^. H_2_S derived from gut microbiota such as *Desulfovibrionaceae* is associated with gastrointestinal disorders, such as UC, Crohn’s disease, and irritable bowel syndrome^56^. Increased levels of *Desulfovibrionaceae* possibly play a role in the pathogenesis of depression via H_2_S-induced inflammation. In addition, levels of fecal H_2_S were higher in UC patients than in healthy control subjects^59,60^. Therefore, it is of great interest to measure the levels of fecal H_2_S in MDD patients. Indeed, further studies on the role of H_2_S-producing bacteria, such as *Desulfovibrionaceae*, in depression are needed.

The genus *Butyricimonas* are butyrate producers with anti-inflammatory properties. We observed decreased levels of *Butyricimonas* in CSDS-susceptible mice, and altered levels of *Butyricimonas* were reported in MDD patients and untreated multiple sclerosis patients^36,61^. *Butyricimonas* produce butyrate, which reduces inflammation and helps maintain a healthy gut. Therefore, decreased levels of *Butyricimonas* in the gut may play an inflammation-based role in the pathogenesis of depression. Interestingly, increase in the levels of *Butyricimonas* induced by (*R*)-ketamine was more potent than that by (*S*)-ketamine. Taken together, the improvement in the levels of *Butyricimonas* may explain the better antidepressant effects of (*R*)-ketamine than (*S*)-ketamine. Considering the possible role of the gut microbiota in the antidepressant actions of certain compounds^42,45^, it is likely that the gut microbiota–brain axis plays at least a partial role in robust antidepressant actions of (*R*)-ketamine.

The precise mechanisms underlying antidepressant actions of ketamine and (*R*)-ketamine are not fully understood. It has been suggested that anti-inflammatory actions of ketamine might play a role in its antidepressant effects^62–65^. Given the role of gut microbiota in the immune system^33–35,42,66^, it is likely that anti-inflammatory action of (*R*)-ketamine might play a role in its antidepressant effects through the immunomodulation by the gut microbiota. Nonetheless, further studies on the anti-inflammatory and antidepressant actions of (*R*)-ketamine in the gut microbiota–brain axis will be needed.

The present data do not provide direct evidence of the effect of gut microbiota on the antidepressant actions of (*R*)-ketamine because behavioral experiments using germ-free mice were not performed. However, it is well known that the antimicrobial effects of currently available antidepressants are important for the correction of the intestinal dysbiosis observed in MDD patients^45^. Therefore, the gut microbiota–brain axis possibly plays a role in the antidepressant actions of (*R*)-ketamine. Nonetheless, additional studies elucidating the relationship between the gut microbiota–brain axis and the antidepressant actions of (*R*)-ketamine are needed. In addition, further studies using germ-free mice will be needed to confirm the role of gut microbiota in the antidepressant effects of (*R*)-ketamine.

In conclusion, the present study suggests that the gut microbiota–brain axis at least partially mediates the antidepressant actions of (*R*)-ketamine. Furthermore, it is likely that the specific effect of (*R*)-ketamine on the decreased levels of *Mollicutes* and *Butyricimonas* after CSDS may explain its robust antidepressant action.
